# Is It Feasible to Perform Infant CPR during Transfer on a Stretcher until Cannulation for Extracorporeal CPR? A Randomization Simulation Study

**DOI:** 10.3390/children11070865

**Published:** 2024-07-17

**Authors:** Myriam Santos-Folgar, Felipe Fernández-Méndez, Martín Otero-Agra, Roberto Barcala-Furelos, Antonio Rodríguez-Núñez

**Affiliations:** 1 REMOSS Research Group, Faculty of Education and Sport Sciences, Universidade de Vigo, 36005 Pontevedra, Spain; 2 School of Nursing, Universidade de Vigo, 36001 Pontevedra, Spain; 3 Department of Obstetrics, Complexo Hospitalario of Pontevedra, Sergas, 36001 Pontevedra, Spain; 4 CLINURSID Research Group, Psychiatry Radiology Public Health Nursing and Medicine Department, Universidade de Santiago de Compostela, 15705 Galicia, Spain; 5 Simulation and Intensive Care Unit of Santiago (SICRUS) Research Group, Health Research Institute of Santiago, University Hospital of Santiago de Compostela—CHUS, 15706 Santiago de Compostela, Spain; 6 Collaborative Research Network Orientated to Health Results (RICORS), Primary Care Interventions to Prevent Maternal and Child Chronic Diseases of Perinatal and Developmental Origin, Instituto de Salud Carlos III, 28029 Madrid, Spain; 7 Faculty of Nursing, Universidade de Santiago de Compostela, 15705 Santiago de Compostela, Spain; 8 Paediatric Critical Intermediate and Palliative Care Section, Hospital Clínico Universitario de Santiago de Compostela, Sergas, 15706 Santiago de Compostela, Spain

**Keywords:** infant, cardiopulmonary resuscitation (CPR), extracorporeal cardiopulmonary resuscitation, extracorporeal membrane oxygenation, transport, CPR quality, stretcher CPR

## Abstract

Introduction: Extracorporeal membrane oxygenation (ECMO) improves infant survival outcomes after cardiac arrest. If not feasible at the place of arrest, victims must be transported to a suitable room to perform ECMO while effective, sustained resuscitation maneuvers are performed. The objective of this simulation study was to compare the quality of resuscitation maneuvers on an infant manikin during simulated transfer on a stretcher (stretcher test) within a hospital versus standard stationary resuscitation maneuvers (control test). Methods: A total of 26 nursing students participated in a randomized crossover study. In pairs, the rescuers performed two 2 min tests, consisting of five rescue breaths followed by cycles of 15 compressions and two breaths. The analysis focused on CPR variables (chest compression and ventilation), CPR quality, the rate of perceived exertion and the distance covered. Results: No differences were observed in the chest compression quality variable (82 ± 10% versus 84 ± 11%, *p* = 0.15). However, significantly worse values were observed in the test for ventilation quality on the stretcher (18 ± 14%) compared to the control test (28 ± 21%), with a value of *p* = 0.030. Therefore, the overall CPR quality was worse in the stretcher test (50 ± 9%) than in the control test (56 ± 13%) (*p* = 0.025). Conclusions: Infant CPR performed by nursing students while walking alongside a moving stretcher is possible. However, in this model, the global CPR quality is less due to the low ventilation quality.

## 1. Introduction

The use of extracorporeal membrane oxygenation (ECMO) during cardiac arrest has become more widespread in recent years for a variety of reasons [1]. ECMO is a highly beneficial tool for the treatment of infants with refractory cardiac arrest, and a favorable neurological outcome can be achieved in the majority of survivors, even after prolonged resuscitation [2].

When compared with continuous conventional CPR, extracorporeal cardiopulmonary resuscitation (E-CPR) after refractory cardiac arrest has been associated with better survival outcomes in pediatric victims [2,3,4].

For this reason, in hospitals with suitably trained staff and adequate resources, early E-CPR should be considered in infants for an apparent reversible cause when conventional advanced life support does not lead to spontaneous recovery [5].

Most in-hospital pediatric cardiac arrests (CA) occur in the Pediatric Intensive Care Unit (PICU) [6]. However, they can also occur in other places such as the emergency room [7,8], hospital wards or even radiology rooms [9]. If available, ECMO could be used in all of these cases and during out-of-hospital cardiac arrests as well [10,11].

As usually, ECMO procedures are not available is some hospital areas, such patients must be transported immediately to a suitable room (i.e., the PICU or operating room) in order to perform ECMO [9] while continuous, as effective as possible resuscitation maneuvers are being performed until the start of ECMO [10]. The main reason is that in order for ECMO to have positive results, it is absolutely essential to minimize flow interruption and pauses. High-quality maneuvers must be performed constantly, and especially during transfer [12,13,14].

The general recommendation is to perform CPR in situ with the victim lying on a firm surface [5]. However, in such cases, resuscitating the victim “on the way to the PICU/operating room” to perform E-CPR would be another of the major exceptions to standard, on-site CPR. However, the evidence regarding CPR quality during intra-hospital transfers with infant victims is extremely limited [9].

The hypothesis is that trained personnel are able to maintain CPR quality during an in-hospital transfer of an infant victim. Therefore, the objective of this study is to evaluate the overall quality of resuscitation maneuvers (chest compressions and ventilations) during manual CPR on an infant manikin during simulated in-hospital transfer.

## 2. Methods

### 2.1. Study Design

**Figure 1:**
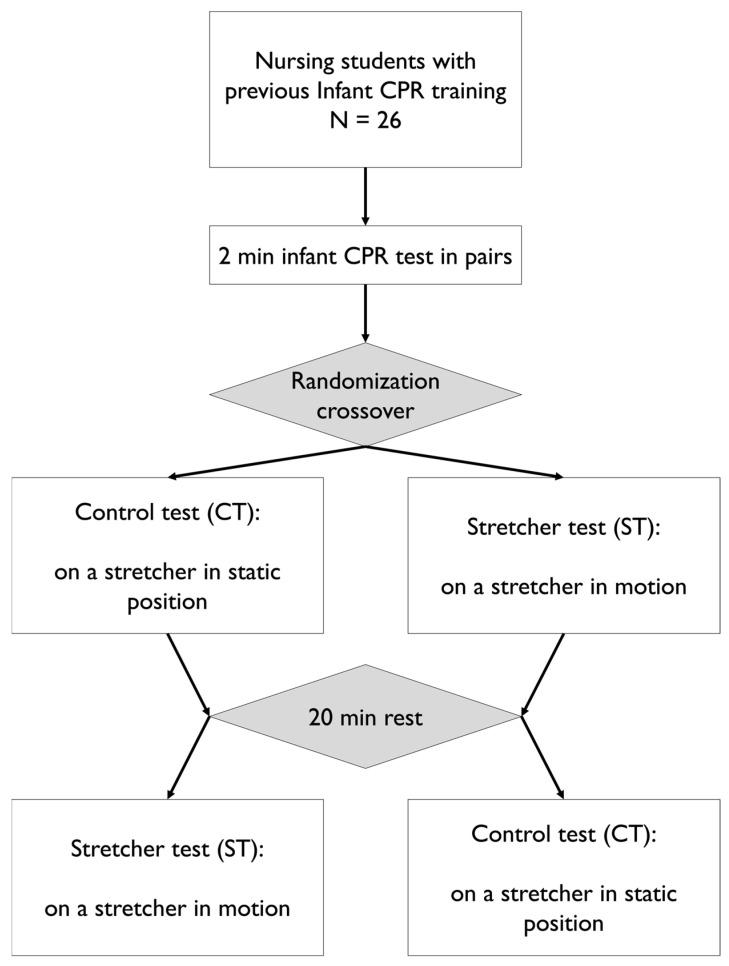
Study design flow chart.

A simulation controlled study was conducted with the aim of comparing CPR during stretcher transfer and baseline CPR by means of a randomized crossover design (Figure 1).

This study involved a convenience sample of 26 nursing students from the Pontevedra School of Nursing (University of Vigo, Pontevedra, Spain). All participants had undergone previous infant CPR training and had duly demonstrated their ability to perform quality CPR (>70%). In addition, the nursing students were familiar with the hospital in question and the material used.

The exclusion criterion comprised not having completed any of the study tests. All participants completed the study and gave informed consent for the use of their data, which were subsequently pseudonymized. This study followed the ethical guidelines of the Declaration of Helsinki. The ethics committee of the University School of Education and Sport Sciences of the University of Vigo, number 19-2802-18, approved the study protocol. This study was conducted in November 2021.

### 2.2. Study Protocol

The details can be seen in Figure 1.

In pairs, the participants randomly performed two 2 min CPR tests on the infant manikin. An emergency scenario was simulated in which an infant victim suffers CA on the hospital ward and is immediately transferred to the operating room in order to perform ECMO. However, in the interim, resuscitation maneuvers are simultaneously being performed (i.e., compressions and ventilations with a self-inflating bag) during the transfer to minimize the risk of fatality.

The rescuers followed the European Resuscitation Council (ERC) 2021 [15] guidelines, performing 5 rescue breaths followed by cycles of 15 compressions (CC) and 2 ventilations (V). One of the participants performed CC via the two-thumb–encircling hands technique. At the same time, the other first responder performed rescue ventilation with the Ambu^®^ SPUR II Infant Resuscitator and Ambu^®^ Baby Face Mask number 0A (a round disposable silicon face mask for patient oxygenation and ventilation) (Ambu, Copenhagen, Denmark). Although a basic CPR infant manikin was used, a real situation was simulated as the manikin was being monitored by the LifePak^®^ 20E Defibrillator/Monitor (Physio-Control, Redmon, WA, USA), a capnography monitor of ventilation and the concentration of CO_2_ exhaled air, and an electrocardiogram. The recorded heart rhythm was non-shockable.

The tests were carried out in a hospital in a randomized order and were made up of the following:

#### 2.2.1. Control Test (CT)

This encompassed baseline CPR with a test in a static position using a manikin on a stretcher without a rigid board.

#### 2.2.2. Stretcher Test (ST) (Figure 2)

This was comprised of a CPR test with an infant manikin carried on a stretcher in an intra-hospital transfer without a rigid board. The subject performing chest compressions stood on the victim’s right hand side and the subject responsible for ventilating was positioned to the left of the victim. Two other emergency staff directed the stretcher. The manikin was placed with its head in the direction of the way forward. The participants completed a predefined 2 min hospital route that consisted of the following:

(A) Passing through a corridor on the ground floor.

(B) Entering and staying in the elevator.

(C) Continuing down the corridor (Figure 1).

It took the subjects 2 min to cover the necessary distance simulated in this study, which is deemed to be sufficient, based on theoretical calculations previously obtained from the Hospital Provincial de Pontevedra, Spain, the Hospital Clínico Universitario de Santiago, Spain, and the Hospital Universitario Policlinico Umberto I in Rome, Italy. However, the little data provided from real pediatric patients suggested that the distance covered was significantly more (taking an average of 7 min) [9]. The distance covered was measured in meters using a tape measure at the end of the route.

No resuscitator changes were made during CPR testing and the subjects were not allowed to see any feedback data. Between each test, a minimum rest period of 20 min was allowed so as to avoid the influence of fatigue on the study. The rating of perceived exertion (RPE) was measured on a 0–10 scale using the Borg scale [16].

**Figure 2:**
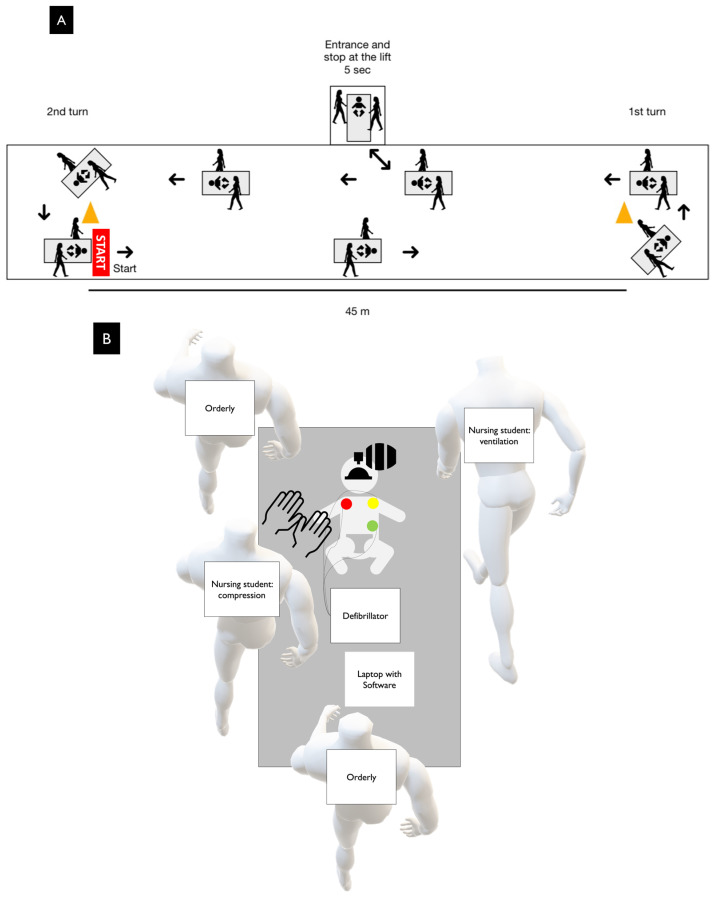
(**A**) A visual diagram of the stretcher test process. (**B**) A diagram of the placement of the materials, participants and assistants of the stretcher test.

### 2.3. Variables and Measuring Equipment

The Laerdal Resusci Baby QCPR^®^ (Stavanger, Norway) was used with the Laerdal Resusci Anne Skillreporter^®^ software version 2.0.0.14 (Stavanger, Norway). The latter is a recording system for determining CPR variables. The manikin corresponds to a 3-month-old baby weighing 5.5 kg [17] and it was configured in accordance with the ERC 2021 recommendations [15]. The following parameters were set for the manikin: a compression ratio between 100 and 120/minute, the correct compression depth in a 36–44 mm range and the correct ventilation volume in a range of 6–10 mL/kg [18,19,20], between 35 and 55 mL.

Several variables were collected in relation to CPR (compressions and ventilations), the quality of CPR, and non-CPR variables (self-perceived fatigue and distance covered).

#### 2.3.1. CPR Variables

Chest compression (CC): number of CC; mean depth in mm; mean rate in CC/min; CC with adequate release as a percentage; CC with adequate hand position as a percentage.

Ventilations (V): number of total V; number of effective V; mean volume in ml.

#### 2.3.2. Quality CPR Variables

Quality parameters were evaluated and disaggregated into CC quality, V quality and CPR quality. Each variable was expressed as a percentage and its calculation is based on the following formulas published in previous studies [20,21,22].

CC quality is calculated using the formula (CC with adequate depth + CC with adequate release + CC with adequate rate) ÷ 3.

V quality is calculated using the formula Number of V with adequate volume/Number of total V × 100.

CPR quality is calculated using the formula (CC quality + V quality)/2.

### 2.4. Statistical Analysis

The sample size was based on an assumed minimum of an effect size of 0.5, an alpha error probability of 0.05 and a statistical power of 0.80. These assumptions provided a sample size of 26 study participants computed by G*Power 3.1.9.2 software (Heinrich Heine University, Düsseldorf, Germany). All statistical analyses were performed using SPSS for Windows, version 21 (SPSS Inc., IBM, Armonk, NY, USA). The quantitative variables were described with measures of central tendency (median) and dispersion (IQR: interquartile range). The qualitative variables were described with absolute and relative frequencies. The quality and distance covered variables were also described with measures of central tendency (mean), dispersion (SD: standard deviation) and confidence estimators (95% CI: 95% confidence interval). For comparisons, the Shapiro–Wilk test was used first in order to check the normality of the data. For parametric variables, the repeated measures Student’s t test for related samples was used, and for non-parametric variables, the comparisons were analyzed using the Wilcoxon signed-rank test. The effect size (ES) was calculated in significance comparisons with Cohen’s d test (parametric variables) and Rosenthal’s r test (non-parametric variables). The following classification was used for the ES: <0.2: trivial/0.2–0.5: small/0.5–0.8: moderate/0.8–1.3: large/>1.3: very large. A significance value of 0.05 was established in all tests.

## 3. Results

### 3.1. Demographic Variables

Table 1 shows the demographic variables of the participants. Of the 26 participants, 77% (n = 20) were women. The median age was 21 years (IQR: 21–24), the median weight was 65 kg (IQR: 58–81) and the median height was 170 cm (IQR: 160–174).

### 3.2. Chest Compression and Ventilation Variables

Table 2 shows the variables that reflect the results of chest compressions and ventilations during the tests. The mean depth was lower in the ST (41 mm; IQR: 40–42) than in the CT (42 mm; IQR: 41–43), with a *p* value = 0.008 and an ES = 0.37. No differences were observed in the mean rate variable (ST: 109 CC/min, IQR: 95–100; CT: 110 CC/min, IQR: 101–115), with a value of *p* = 0.92. The ST presented a lower percentage of CC with adequate release (ST: 82%, IQR: 63–92 compared to CT: 99%, IQR: 91–100), with a *p* value < 0.001 and an ES = 0.52. No differences were observed in CC with adequate hand position (*p* > 0.05). Regarding ventilations, the number of total V was 20 V (IQR: 18–20) in the ST vs. 18 V (IQR: 18–19) in the CT, with *p* = 0.010 and IS = 0.36. Despite performing a greater number of ventilation attempts in the ST, the number of effective V was lower compared to the CT (ST: 14 V, IQR: 10–16; CT: 18 V, IQR: 16–18), with a *p* value = 0.002 and an ES = 0.93. On the other hand, the mean volume did not show any differences between the tests (*p* = 0.45).

**Figure 3:**
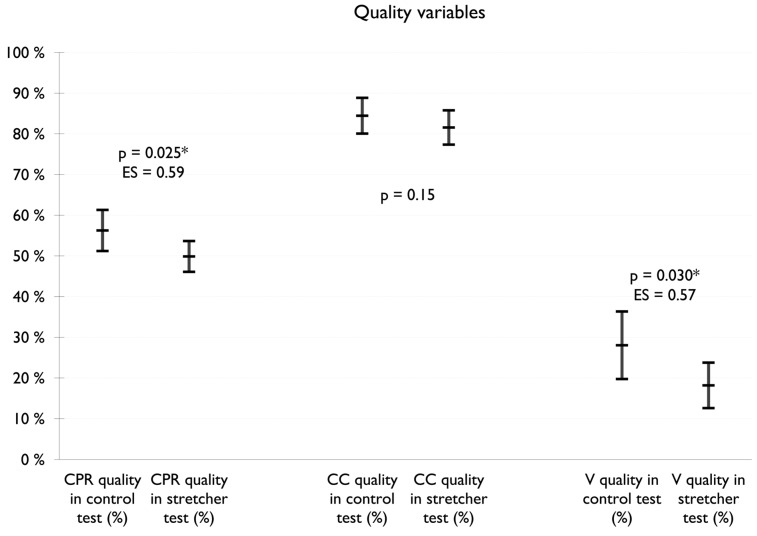
Results of the quality variables. * Statistically significant.

Figure 3 shows the results of the quality variables. No differences were observed in the CC quality variable: the ST presented a mean of 82% (SD: 10 and 95% CI: 77–86) and the CT presented a mean of 84% (SD: 11 and 95% CI: 80–89) with a value of *p* = 0.15. However, significantly worse values were observed in V quality in the ST test, with a mean of 18% (SD: 14 and 95% CI: 13–24) compared to the CT, which had a mean of 28% (SD: 21 and 95% CI: 20–36) with a *p* value = 0.030 and an ES = 0.57. These differences in the quality of the ventilations were relevant to the CPR quality, in which significantly lower values were also observed in the ST, with a mean of 50% (SD: 9 and 95% CI: 46–54), than in the CT, with a mean of 56% (SD: 13 and 95% CI: 51–61), with a *p* value = 0.025 and an ES = 0.59.

**Figure 4:**
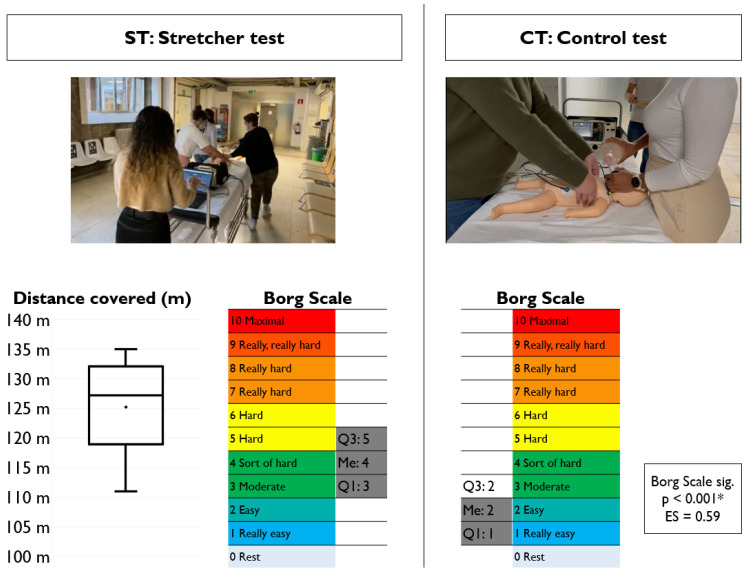
The results of the variables of perception of fatigue and distance covered. Images of each of the tests. * Statistically significant.

Figure 4 shows the results for the non-CPR variables. The mean distance covered in the ST was 125 m (SD: 7 and 95% CI: 122–128). On the other hand, perceived fatigue assessed through the Borg scale was significantly higher in the ST (4; IQR: 3–5) than in the CT (2; IQR: 1–2), with a *p* value <0.001 and an ES = 0.59.

## 4. Discussion

In the event of an in-hospital pediatric CA requiring ECMO but out of the PICU or operating room, effective resuscitation maneuvers should start and continue while transferring the infant to a suitable room to perform E-CPR. It is essential to perform quality resuscitative maneuvers while transferring a pediatric casualty to perform E-CPR.

The main objective of this study was to evaluate the quality of manual CPR during intra-hospital transport of the infant manikin on a stretcher.

The main positive finding of this investigation was that the quality of CPR and chest compressions, in particular, can be maintained while transferring the pediatric victim (in our case, an infant manikin) on a stretcher. On the other hand, a negative finding was that despite being properly trained, the participants failed to perform high-quality ventilations, neither standing up nor walking. The poor performance therefore makes it impossible to know if it is actually the transfer itself which deteriorates the ventilation quality or failure of their skills training.

Chest compression quality is essential for improving CPR results [23]. Several investigations have shown that CPR with high-quality chest compressions and a high chest compression fraction is associated with increased survival [24,25]. Our results show that compression quality was generally high in both simulations (84% CT vs. 82% ST) and no difficulties were noted when they were carried out while walking next to the stretcher, with values similar to other studies involving infants [20,26]. The results of our investigation agree with those of Cheskes et al. who reported that pre-hospital rescuers can perform high-quality manual compressions during transport in out-of-hospital cardiac arrest [27]. Similarly, Loaec et al. demonstrated with an observational study that it is possible to maintain CPR quality during the intra-hospital transport of critically ill infants with chest compression rates and depths that met international recommendations [9].

The nursing students that participated in this study performed adequate compressions during transport. Our data confirm that trained rescuers were able to perform quality compressions on the move.

Aufderheide et al. observed that incomplete chest recoil during CPR was frequent in real patients in out-of-hospital cardiac arrest [28], which is consistent with the results of our investigation and is therefore an important point that needs to be improved. One explanation for incomplete chest recoil may be the compression technique used, since various investigations have noted that the two-thumb–encircling hands technique is related to a higher rate of incomplete recoil [29,30]. Several investigations are currently focused on implementing new compression methods in infant victims [26,31,32], although they are still in the experimental phase, so it is possible that these new techniques could improve this aspect over time.

Suitable ventilation is also fundamental for success during CPR in a pediatric victim, since the most frequent cause of cardiac arrest in infants is respiratory failure [15,33]. In our investigation, we observed low ventilation quality in the control test and a significant decrease in ventilation quality during intra-hospital transfer. This coincides with Lipman et al.’s investigation which showed a decrease in ventilation volume during intra-hospital transport in a simulated maternal cardiac arrest [34].

Similarly, we observed suboptimal ventilation quality values in both tests, with lower values during pediatric intra-hospital transfer (18% ST vs. 28% CT). In the same way, other investigations observed that the instrumental management of the pediatric airway is difficult even for health professionals [21,22,35,36]. Although we have chosen bag-mask ventilation as the more realistic initial airway management in cases of at-ward cardiac arrest, supported by relevant guidelines, we could speculate about the impact of supraglottic devices on the quality of ventilations. Regardless, it is essential to practice this aspect thoroughly during training and perform rolling refreshers during work activity [37].

Despite the greater number of attempts with the stretcher test, more ventilations were performed in the control test, which implies that ventilation management is hampered due to the stretcher being in motion. This is the main obstacle to overcome and emergency staff need to work on this aspect more in the future in order to improve this critical part of the emergency procedure. So, in a situation where there is a need to transport an infant who is on standby and who does not have an isolated airway, the key question is the following: Is it worth stopping in order to isolate the airway and hence delay an important transfer (e.g., for ECMO)? Or should we transfer the infant directly for ECMO while simultaneously performing CPR and thus delay isolating the airway until we perform ECMO?

Recent research has shown that instrumental airway management is associated with higher chances of survival compared to advanced management [38,39]. Pediatric endotracheal intubation is a complex procedure which is performed infrequently and requires ongoing skill maintenance to be successful [40]. In addition, pediatric intubation mistakes are commonplace [41]. For this reason, ventilation with a self-inflating bag mask has become the essential technique for performing ventilations [42]. The recommendation is not to secure the airway but rather to oxygenate and ventilate. The low-quality results of the ventilations in this investigation cannot be attributed to the victim not being intubated. One strategy to improve ventilation could be to perform breaths involving both rescuers: one seals the mask to the victim’s face and opens the airway while the other rescuer squeezes the bag to blow air into the lungs [42].

The performance of high-quality CPR is an important determinant of survival with a good neurological outcome after cardiac arrest [36]. The total quality of CPR decreased slightly (56% CT vs. 50% ST) during stretcher transfer, mainly due to the ventilatory component. The suboptimal values noted in both situations coincide with Ødegaard et al.’s results. They showed that CPR quality in adult patients did not deteriorate during ambulance transport, but that it was suboptimal both in the static situation and during transport [43].

The only study that analyzed the quality of CPR in real pediatric patients during intra-hospital transfer on a stretcher showed that CPR quality was maintained during transport, so they suggest incorporating transport to facilitate ECMO cannulation [9].

Resuscitation maneuvers are physically demanding even for trained rescuers [44]. The Borg scale is frequently used in research studies to estimate the magnitude of perceived exertion during CPR [20,45,46]. It is perfectly logical that fatigue is greater during intra-hospital transport than during static CPR. However, fatigue during transport did not reach crucial values and the normal and sustainable values seen in the tests were similar to those observed in other investigations [20,26].

During the 2 min of CPR, the entrance, exit and stay in the elevator were included, since this is common during the intra-hospital transfer of a victim. This is a weak point of this study as elevator times could be longer in other hospitals. The entrance and exit from the elevator were critical moments in the transfer of the patient in cardiorespiratory arrest due to the number of personnel who were involved in the transfer and the narrowness of the elevator. So, it becomes a situation in which we must train personnel properly and describe it clearly in the protocols in order to improve performance.

This study has a number of limitations that should be mentioned. On the one hand, the study was carried out in a simulated environment, so the results cannot be extrapolated to clinical practice. On the other hand, elevator times could be longer in other hospitals. Also, we have no comparison of different airway management strategies (mask, supraglottic devices, intratracheal tube).

## 5. Conclusions

Infant in-hospital CPR with stretcher transport involving two rescuers and two attendants is viable and feasible. However, students with previous training in infant CPR perform it with reduced quality during transport on a stretcher, mainly due to the difficulty of ventilation. It is necessary to look for new strategies to improve CPR quality during the transport of a pediatric victim on a stretcher. Another limitation is the presence of distracting factors that may have influenced the results. These factors include participant fatigue, the simulated environment, and psychological stressors.

## Figures and Tables

**Table 1:** Demographics.

Variables	Total (n = 26)	
	Me	IQR
Age (in years)	21	(21–24)
Weight (in kg)	65	(58–81)
Height (in cm)	170	(160–174)
	**n**	**(%)**
Sex		
Women	20	(77%)
Men	6	(23%)

Me: median. IQR: interquartile range (Q1–Q3). n: absolute frequency. (%): relative frequency.

**Table 2:** Chest compressions and ventilations (n = 26).

Variables	Control Test		Stretcher Test		Significance
	Me	IQR	Me	IQR	
CC: Chest Compression					
Number of CC	143	(136–156)	153	(147–159)	*p* = 0.031 (0.46) *
Mean depth (mm)	42	(41–43)	41	(40–42)	*p* = 0.008 (0.37) †
Mean rate (CC/min)	109	(99–115)	110	(101–115)	*p* = 0.92 *
CC with adequate release (%)	99	(91–100)	82	(63–92)	*p* < 0.001 (0.52) †
CC with adequate hand position (%)	100	(80–100)	100	(97–100)	*p* = 0.24 †
V: Ventilation					
Number of total V	18	(18–19)	20	(18–20)	*p* = 0.010 (0.36) †
Number of effective V	18	(16–18)	14	(10–16)	*p* = 0.002 (0.93) *
Mean volume (mL)	48	(29–60)	47	(35–58)	*p* = 0.45 †

Me: median. IQR: interquartile range (Q1-Q3). * Repeated measures Student’s t test (*p* = 0.05). In brackets: effect size (Cohen’s d test). † Wilcoxon signed-rank test (*p* = 0.05). In brackets: effect size (Rosenthal’s r test). Effect size: (<0.2: trivial/0.2–0.5: small/0.5–0.8: moderate/0.8–1.3: large/>1.3: very large).

## Data Availability

The original contributions presented in the study are included in the article, further inquiries can be directed to the corresponding author.
